# (*E*)-2-[4-(Dimethyl­amino)styr­yl]-1-methyl­quinolinium 4-methoxy­benzene­sulfonate monohydrate^1^

**DOI:** 10.1107/S1600536808005205

**Published:** 2008-02-29

**Authors:** Thawanrat Kobkeatthawin, Pumsak Ruanwas, Suchada Chantrapromma, Hoong-Kun Fun

**Affiliations:** a Department of Chemistry, Faculty of Science, Prince of Songkla University, Hat-Yai, Songkhla 90112, Thailand; b X-ray Crystallography Unit, School of Physics, Universiti Sains Malaysia, 11800 USM, Penang, Malaysia

## Abstract

In the title compound, C_20_H_21_N_2_^+^·C_7_H_7_O_4_S^−^·H_2_O, the cation is nearly planar and exists in the *E* configuration. The cations and anions form individual chains along the *b* axis and are inter­connected by weak C—H⋯O inter­actions. The 4-methoxy­benzensulfonate anions are linked to water mol­ecules through O—H⋯O hydrogen bonds, forming a three-dimensional network. The crystal structure is further stabilized by a C—H⋯π inter­action involving the methoxy­phenyl ring. The sulfonate anion is also involved in a weak intra­molecular C—H⋯O inter­action which generates an *S*(5) ring motif.

## Related literature

For bond lengths and angles, see: Allen (2002 ▶); Allen *et al.* (1987 ▶). For details of hydrogen-bond motifs, see: Bernstein *et al.* (1995 ▶). For background to NLO materials research, see: Chia *et al.*, (1995 ▶); Otero *et al.*, (2002 ▶). For related structures, see for example: Chantrapromma *et al.* (2006 ▶, 2007 ▶, 2007*a* ▶,*b* ▶); Jindawong *et al.* (2005 ▶); Dittrich *et al.* (2003 ▶); Nogi *et al.* (2000 ▶); Sato *et al.* (1999 ▶).
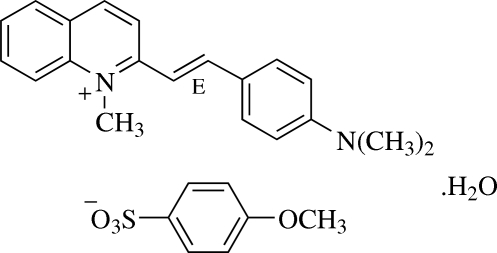


## Experimental

### 

#### Crystal data

C_20_H_21_N_2_^+^·C_7_H_7_O_4_S^−^·H_2_O
*M* *_r_* = 494.60
Monoclinic, 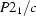

*a* = 14.6064 (5) Å
*b* = 10.4253 (4) Å
*c* = 19.5025 (6) Å
β = 126.737 (2)°
*V* = 2379.94 (16) Å^3^
*Z* = 4
Mo *K*α radiation
μ = 0.18 mm^−1^
*T* = 100.0 (1) K
0.58 × 0.27 × 0.19 mm

#### Data collection

Bruker SMART APEX2 CCD area-detector diffractometer
Absorption correction: multi-scan (*SADABS*; Bruker, 2005 ▶) *T*_min_ = 0.904, *T*_max_ = 0.967
33983 measured reflections
6953 independent reflections
5445 reflections with *I* > 2σ(*I*)
*R*_int_ = 0.045

#### Refinement

*R*[*F* ^2^ > 2σ(*F* ^2^)] = 0.052
*wR*(*F* ^2^) = 0.139
*S* = 1.06
6953 reflections
350 parameters
H-atom parameters constrained
Δρ_max_ = 0.74 e Å^−3^
Δρ_min_ = −0.42 e Å^−3^

### 

Data collection: *APEX2* (Bruker, 2005 ▶); cell refinement: *APEX2*; data reduction: *SAINT* (Bruker, 2005 ▶); program(s) used to solve structure: *SHELXTL* (Sheldrick, 2008 ▶); program(s) used to refine structure: *SHELXTL*; molecular graphics: *SHELXTL*; software used to prepare material for publication: *SHELXTL* and *PLATON* (Spek, 2003 ▶).

## Supplementary Material

Crystal structure: contains datablocks global, I. DOI: 10.1107/S1600536808005205/sj2466sup1.cif

Structure factors: contains datablocks I. DOI: 10.1107/S1600536808005205/sj2466Isup2.hkl

Additional supplementary materials:  crystallographic information; 3D view; checkCIF report

## Figures and Tables

**Table 1:** Hydrogen-bond geometry (Å, °)

*D*—H⋯*A*	*D*—H	H⋯*A*	*D*⋯*A*	*D*—H⋯*A*
O1*W*—H1*W*⋯O2^i^	0.84	2.04	2.875 (3)	169
O1*W*—H2*W*⋯O4^ii^	0.85	2.10	2.926 (2)	161
C7—H7*A*⋯O3^iii^	0.93	2.49	3.015 (3)	116
C8—H8*A*⋯O3^iii^	0.93	2.57	3.049 (3)	113
C20—H20*A*⋯O4^iv^	0.96	2.46	3.325 (2)	151
C23—H23*A*⋯O1*W*^v^	0.93	2.44	3.365 (2)	176
C26—H26*A*⋯O4	0.93	2.56	2.921 (2)	104
C27—H27*A*⋯O1*W*^vi^	0.96	2.58	3.160 (3)	119
C27—H27*A*⋯O1^vii^	0.96	2.55	3.282 (2)	133
C16—H16*A*⋯*Cg*1^iv^	0.93	2.81	3.6513 (19)	151

Symmetry codes: (i) 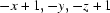; (ii) 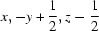; (iii) 
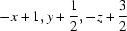
; (iv) 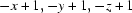; (v) 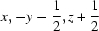; (vi) 
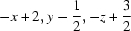
; (vii) 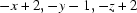. *Cg*1 is the centroid of the C21–C26 methoxy­phenyl ring.

**Fig. 1.**
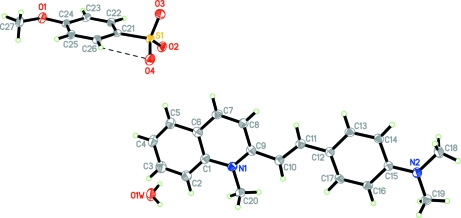
The asymmetric unit of (I) showing 50% probability displacement ellipsoids and the atom-numbering scheme. The weak intramolecular C—H···O interaction is drawn as a dashed line.

**Fig. 2.**
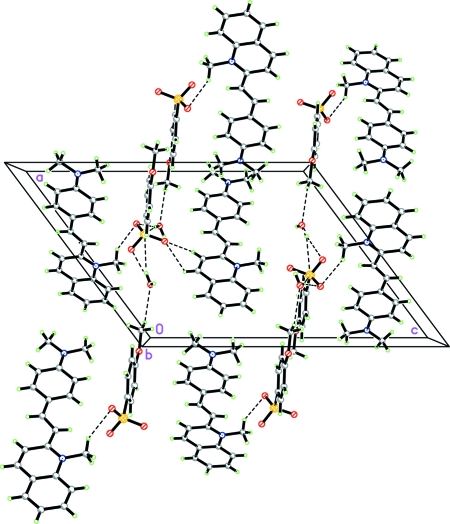
The crystal packing of (I) viewed along the b axis. The O—H···O and weak C—H···O interactions are drawn as dashed lines.

## supplementary crystallographic information

### Comment

A lot of research have been done to search for second-order nonlinear optic (NLO) materials. Organic crystals with the required conjugated π electrons are attractive candidates because of their large NLO coefficients (Chia *et al.*, 1995; Dittrich *et al.*, 2003; Otero *et al.*, 2002; Nogi *et al.*, 2000; Sato *et al.*, 1999). In our research on this kind of materials, we have previously synthesized and crystallized several organic ionic salts of quinolinium derivatives to study their non-linear optical properties (Chantrapromma *et al.*, 2006; 2007*a*,b; 2007; Jindawong *et al.*, 2005). Previous studies (Dittrich *et al.*, 2003; Nogi *et al.*, 2000; Sato *et al.*, 1999) have shown that the 1-methyl-4-(2-(4-(dimethylamino)phenyl)ethynyl)pyridinium *p*-toluenesulfonate (DAST) and its analogues exhibit second-order non-linear optical properties. Based on these previous studies, we have synthesized the title compound which was designed to increase the π-conjugation in the system with the replacement of the cationic 4-hydroxy-3-methoxyphenyl ring that is present in 2-[(*E*)-(4-Hydroxy-3-methoxyphenyl)ethenyl]-1-methylquinolinium 4-methoxybenzenesulfonate (Chantrapromma *et al.*, 2007*a*) by the 4-dimethylaminophenyl ring. The synthesis and crystal structure of the title compound, (I), Fig 1, are reported in this study. Unfortunately this crystal does not have second-order NLO properties because it crystallized out in a centrosymmetric space group.

The asymmetric unit of the title compound consists of the C_20_H_21_N_2_^+^ cation, C_7_H_7_O_4_S^-^ anion and one H_2_O molecule. The cation exists in the *E* configuration with respect to the C10?C11 double bond [1.350 (2) Å] and is nearly planar as indicated by the dihedral angle between the quinolinium and the dimethylaminophenyl rings being 3.41 (7)° and the torsion angles C8–C9–C10–C11 = -8.7 (2)° and C10–C11–C12–C17 = 3.2 (3)°. The relative arrangement of cation and anion is shown by the angles between the mean plane of the methoxyphenyl ring and those of the quinolinium and dimethylaminophenyl systems which are 81.29 (7)° and 78.29 (8)°, respectively.

The atom O4 of the sulfonate contributes to a weak intramolecular C—H···O interaction (Fig. 1 and Table 1) forming an S(5) ring motif (Bernstein *et al.*, 1995). The bond lengths and angles are normal (Allen *et al.*, 1987) and are comparable with closely related structures (Chantrapromma *et al.*, 2006; 2007*a*; 2007*b*; 2007*c*).

In the crystal packing, the O2 and O4 atoms of 4-methoxybenzenesulfonate anion are involved in the O—H···O hydrogen bonds whereas O3 and O4 atoms are involved in weak C—H···O interactions (Table 1). The cations and anions form individual chains along the *b* axis and are interconnected by weak C—H···O interactions. The 4-methoxybenzensulfonate anions are linked to water molecules through O—H···O hydrogen bonds forming a three dimensional network (Fig. 2). The crystal structure is further stabilized by a C16—H16A···π interaction to the methoxyphenyl ring [C21–C26]: C16—H16A=0.93; H16A···*Cg*^i^=2.8096; C16—*Cg*_1_^i^=3.6513 (19) Å; C16—H16A···*Cg*_1_^i^= 151°. [*Cg*_1_^i^ is the centroid of the C21–C26 methoxyphenyl ring (symmetry code: (i): 1 - *x*, 1 - *y*, 1 - *z*).]

### Experimental

2-(4-dimethylaminostyryl)-1-methylquinolinium iodide (compound A) was synthesized by mixing a solution (1:1:1 molar ratio) of 1,2-dimethylquinolinium iodide (2.00 g, 7.01 mmol), dimethylaminobenzaldehyde (1.05 g, 7.01 mmol) and piperidine (0.70 g, 7.01 mmol) in hot methanol (50 ml). The resulting solution was refluxed for 6 h under a nitrogen atmosphere. The resultant solid was filtered off, washed with methanol and recrystallized from methanol to give green crystals of compound A. Silver(I) 4-methoxybenzenesulfonate (compound B) was synthesized according to our previously reported procedure (Chantrapromma *et al.*, 2007*a*). The title compound was synthesized by mixing compound A (0.2 g, 0.48 mmol) in hot methanol (50 ml) and compound B (0.14 g, 0.48 mmol) in hot methanol (20 ml). The mixture immediately yielded a grey precipitate of silver iodide. After stirring the mixture for *ca* 30 min, the precipitate was removed and the resulting solution was evaporated yielding a brown solid. Brown block-shaped single crystals of the title compound suitable for *x*-ray structure determination were recrystalized from methanol/ethanol solvent (3:1 *v*/*v*) by slow evaporation of the solvent at room temperature after a few weeks. (Mp. 545–547 K).

### Refinement

All H atoms were placed in calculated positions with d(O—H) = 0.85 Å, *U*_iso_=1.2*U*_eq_(O), d(C—H) = 0.93 Å, *U*_iso_=1.2*U*_eq_(C) for aromatic and CH, 0.96 Å, *U*_iso_ = 1.5*U*_eq_(C) for CH_3_ atoms. A rotating group model was used for the methyl groups. The highest residual electron density peak is located at 0.88 Å from C9 and the deepest hole is located at 0.69 Å from S1.

### Crystal data

**Table:**

C_20_H_21_N_2_^+^·C_7_H_7_O_4_S^–^·H_2_O	*F*_000_ = 1048
*M**_r_* = 494.60	*D*_x_ = 1.380 Mg m^−^^3^
Monoclinic, *P*2_1_/*c*	Melting point = 545–547 K
Hall symbol: -P 2ybc	Mo *K*α radiation λ = 0.71073 Å
*a* = 14.6064 (5) Å	Cell parameters from 6593 reflections
*b* = 10.4253 (4) Å	θ = 2.1–30.0º
*c* = 19.5025 (6) Å	µ = 0.18 mm^−^^1^
β = 126.737 (2)º	*T* = 100.0 (1) K
*V* = 2379.94 (16) Å^3^	Block, brown
*Z* = 4	0.58 × 0.27 × 0.19 mm

### Data collection

**Table:**

Bruker SMART APEX2 CCD area-detector diffractometer	6953 independent reflections
Radiation source: fine-focus sealed tube	5445 reflections with *I* > 2σ(*I*)
Monochromator: graphite	*R*_int_ = 0.045
Detector resolution: 8.33 pixels mm^-1^	θ_max_ = 30.0º
*T* = 100.0(1) K	θ_min_ = 2.1º
ω scans	*h* = −20→19
Absorption correction: multi-scan(SADABS; Bruker, 2005)	*k* = −14→14
*T*_min_ = 0.904, *T*_max_ = 0.967	*l* = −24→27
33983 measured reflections	

### Refinement

**Table:**

Refinement on *F*^2^	Secondary atom site location: difference Fourier map
Least-squares matrix: full	Hydrogen site location: inferred from neighbouring sites
*R*[*F*^2^ > 2σ(*F*^2^)] = 0.052	H-atom parameters constrained
*wR*(*F*^2^) = 0.139	*w* = 1/[σ^2^(*F*_o_^2^) + (0.0641*P*)^2^ + 1.1852*P*] where *P* = (*F*_o_^2^ + 2*F*_c_^2^)/3
*S* = 1.06	(Δ/σ)_max_ = 0.001
6953 reflections	Δρ_max_ = 0.75 e Å^−^^3^
350 parameters	Δρ_min_ = −0.42 e Å^−^^3^
Primary atom site location: structure-invariant direct methods	Extinction correction: none

### Special details

**Table:**

Experimental. The low-temparture data was collected with the Oxford Cryosystem Cobra low-temperature attachment.
Geometry. All e.s.d.'s (except the e.s.d. in the dihedral angle between two l.s. planes) are estimated using the full covariance matrix. The cell e.s.d.'s are taken into account individually in the estimation of e.s.d.'s in distances, angles and torsion angles; correlations between e.s.d.'s in cell parameters are only used when they are defined by crystal symmetry. An approximate (isotropic) treatment of cell e.s.d.'s is used for estimating e.s.d.'s involving l.s. planes.
Refinement. Refinement of *F*^2^ against ALL reflections. The weighted *R*-factor *wR* and goodness of fit *S* are based on *F*^2^, conventional *R*-factors *R* are based on *F*, with *F* set to zero for negative *F*^2^. The threshold expression of *F*^2^ > σ(*F*^2^) is used only for calculating *R*-factors(gt) *etc*. and is not relevant to the choice of reflections for refinement. *R*-factors based on *F*^2^ are statistically about twice as large as those based on *F*, and *R*- factors based on ALL data will be even larger.

### Fractional atomic coordinates and isotropic or equivalent isotropic displacement parameters (Å^2^)

**Table:**

	*x*	*y*	*z*	*U*_iso_*/*U*_eq_	
S1	0.61826 (3)	0.05907 (4)	0.76956 (2)	0.02112 (11)	
O1	0.95438 (10)	−0.35916 (12)	0.95862 (7)	0.0250 (3)	
O2	0.53003 (10)	0.00579 (13)	0.68519 (7)	0.0275 (3)	
O3	0.57692 (12)	0.08820 (15)	0.81913 (8)	0.0360 (3)	
O4	0.67900 (11)	0.16597 (13)	0.76497 (8)	0.0301 (3)	
O1W	0.66931 (11)	0.09083 (15)	0.33524 (10)	0.0429 (4)	
H1W	0.6058	0.0660	0.3224	0.058 (8)*	
H2W	0.6633	0.1682	0.3187	0.050 (7)*	
N1	0.56771 (11)	0.67419 (14)	0.49475 (8)	0.0215 (3)	
N2	0.05342 (12)	1.30700 (15)	0.29537 (8)	0.0235 (3)	
C1	0.64527 (13)	0.57400 (17)	0.54367 (9)	0.0217 (3)	
C2	0.70847 (15)	0.51470 (19)	0.52077 (11)	0.0277 (4)	
H2A	0.7021	0.5417	0.4726	0.038 (6)*	
C3	0.77995 (16)	0.4162 (2)	0.56969 (11)	0.0322 (4)	
H3A	0.8227	0.3772	0.5545	0.055 (7)*	
C4	0.79097 (16)	0.37240 (19)	0.64227 (11)	0.0304 (4)	
H4A	0.8393	0.3040	0.6737	0.043 (6)*	
C5	0.73095 (14)	0.42956 (18)	0.66701 (10)	0.0268 (4)	
H5A	0.7384	0.4009	0.7153	0.024 (5)*	
C6	0.65650 (13)	0.53407 (17)	0.61789 (10)	0.0229 (3)	
C7	0.59337 (14)	0.59648 (18)	0.64128 (10)	0.0237 (3)	
H7A	0.5989	0.5687	0.6889	0.026 (5)*	
C8	0.52447 (14)	0.69699 (18)	0.59453 (10)	0.0241 (3)	
H8A	0.4857	0.7396	0.6120	0.024 (5)*	
C9	0.51057 (13)	0.73831 (17)	0.51874 (9)	0.0216 (3)	
C10	0.43701 (13)	0.84531 (17)	0.46835 (10)	0.0220 (3)	
H10A	0.4384	0.8763	0.4243	0.032 (6)*	
C11	0.36646 (13)	0.90197 (17)	0.48240 (9)	0.0211 (3)	
H11A	0.3685	0.8710	0.5280	0.033 (6)*	
C12	0.28800 (13)	1.00599 (16)	0.43337 (9)	0.0197 (3)	
C13	0.22320 (13)	1.05721 (17)	0.45820 (9)	0.0218 (3)	
H13A	0.2324	1.0239	0.5062	0.039 (6)*	
C14	0.14611 (13)	1.15578 (17)	0.41354 (9)	0.0215 (3)	
H14A	0.1048	1.1876	0.4321	0.029 (5)*	
C15	0.12911 (12)	1.20908 (16)	0.33990 (9)	0.0188 (3)	
C16	0.19398 (13)	1.15699 (17)	0.31446 (9)	0.0202 (3)	
H16A	0.1845	1.1893	0.2661	0.025 (5)*	
C17	0.27101 (13)	1.05893 (17)	0.36003 (9)	0.0206 (3)	
H17A	0.3128	1.0270	0.3419	0.023 (5)*	
C18	−0.01161 (15)	1.3601 (2)	0.32301 (11)	0.0292 (4)	
H18A	0.0396	1.3844	0.3821	0.029 (5)*	
H18B	−0.0642	1.2968	0.3162	0.034 (6)*	
H18C	−0.0532	1.4341	0.2890	0.054 (8)*	
C19	0.03078 (15)	1.35334 (19)	0.21618 (10)	0.0260 (4)	
H19A	0.1000	1.3866	0.2277	0.036 (6)*	
H19B	−0.0257	1.4200	0.1926	0.043 (7)*	
H19C	0.0031	1.2839	0.1760	0.028 (5)*	
C20	0.54745 (16)	0.7057 (2)	0.41323 (11)	0.0288 (4)	
H20A	0.4713	0.7384	0.3738	0.051 (7)*	
H20B	0.5564	0.6299	0.3898	0.044 (7)*	
H20C	0.6014	0.7695	0.4228	0.048 (7)*	
C21	0.72090 (13)	−0.06494 (16)	0.82541 (9)	0.0190 (3)	
C22	0.68657 (13)	−0.19286 (17)	0.81620 (9)	0.0210 (3)	
H22A	0.6094	−0.2136	0.7793	0.024 (5)*	
C23	0.76655 (14)	−0.28880 (17)	0.86144 (9)	0.0218 (3)	
H23A	0.7432	−0.3737	0.8553	0.032 (6)*	
C24	0.88269 (13)	−0.25763 (16)	0.91662 (9)	0.0198 (3)	
C25	0.91782 (13)	−0.13092 (16)	0.92572 (9)	0.0209 (3)	
H25A	0.9950	−0.1102	0.9623	0.030 (5)*	
C26	0.83634 (13)	−0.03516 (17)	0.87967 (9)	0.0207 (3)	
H26A	0.8596	0.0497	0.8853	0.021 (5)*	
C27	1.07377 (14)	−0.33401 (19)	1.02041 (11)	0.0272 (4)	
H27A	1.1131	−0.4129	1.0475	0.024 (5)*	
H27B	1.0848	−0.2755	1.0628	0.031 (5)*	
H27C	1.1035	−0.2968	0.9924	0.038 (6)*	

### Atomic displacement parameters (Å^2^)

**Table:**

	*U*^11^	*U*^22^	*U*^33^	*U*^12^	*U*^13^	*U*^23^
S1	0.02159 (18)	0.0248 (2)	0.01629 (17)	0.00406 (15)	0.01098 (14)	0.00242 (14)
O1	0.0220 (5)	0.0216 (6)	0.0234 (5)	0.0026 (5)	0.0093 (4)	0.0033 (5)
O2	0.0230 (6)	0.0336 (7)	0.0176 (5)	0.0029 (5)	0.0077 (5)	0.0016 (5)
O3	0.0390 (7)	0.0486 (9)	0.0284 (6)	0.0158 (7)	0.0244 (6)	0.0072 (6)
O4	0.0289 (6)	0.0248 (7)	0.0309 (6)	0.0018 (5)	0.0148 (5)	0.0054 (5)
O1W	0.0259 (7)	0.0310 (8)	0.0591 (9)	0.0033 (6)	0.0186 (7)	0.0143 (7)
N1	0.0211 (6)	0.0261 (8)	0.0164 (6)	0.0002 (5)	0.0107 (5)	0.0007 (5)
N2	0.0241 (6)	0.0285 (8)	0.0181 (6)	0.0070 (6)	0.0128 (5)	0.0032 (5)
C1	0.0180 (7)	0.0230 (8)	0.0167 (6)	−0.0035 (6)	0.0065 (5)	−0.0007 (6)
C2	0.0264 (8)	0.0331 (10)	0.0228 (7)	0.0006 (7)	0.0143 (7)	−0.0013 (7)
C3	0.0326 (9)	0.0343 (11)	0.0284 (8)	0.0044 (8)	0.0175 (7)	−0.0010 (7)
C4	0.0309 (9)	0.0274 (10)	0.0274 (8)	0.0069 (7)	0.0145 (7)	0.0042 (7)
C5	0.0231 (7)	0.0299 (10)	0.0210 (7)	0.0015 (7)	0.0097 (6)	0.0015 (7)
C6	0.0194 (7)	0.0258 (9)	0.0205 (7)	−0.0050 (6)	0.0102 (6)	−0.0058 (6)
C7	0.0238 (7)	0.0290 (9)	0.0165 (6)	−0.0025 (7)	0.0109 (6)	−0.0001 (6)
C8	0.0213 (7)	0.0291 (9)	0.0210 (7)	0.0012 (7)	0.0121 (6)	0.0021 (6)
C9	0.0176 (6)	0.0240 (9)	0.0205 (7)	−0.0033 (6)	0.0099 (6)	−0.0048 (6)
C10	0.0205 (7)	0.0253 (9)	0.0177 (6)	0.0007 (6)	0.0101 (6)	−0.0001 (6)
C11	0.0207 (7)	0.0237 (8)	0.0171 (6)	−0.0009 (6)	0.0103 (6)	−0.0007 (6)
C12	0.0179 (6)	0.0217 (8)	0.0173 (6)	−0.0011 (6)	0.0094 (5)	−0.0020 (6)
C13	0.0215 (7)	0.0276 (9)	0.0169 (6)	−0.0001 (6)	0.0117 (6)	0.0012 (6)
C14	0.0211 (7)	0.0278 (9)	0.0174 (6)	0.0014 (6)	0.0126 (6)	−0.0017 (6)
C15	0.0169 (6)	0.0208 (8)	0.0158 (6)	−0.0003 (6)	0.0082 (5)	−0.0020 (5)
C16	0.0202 (7)	0.0256 (8)	0.0160 (6)	−0.0007 (6)	0.0114 (6)	−0.0005 (6)
C17	0.0180 (6)	0.0275 (9)	0.0178 (6)	0.0009 (6)	0.0114 (5)	−0.0027 (6)
C18	0.0274 (8)	0.0354 (10)	0.0247 (8)	0.0099 (8)	0.0156 (7)	0.0014 (7)
C19	0.0251 (8)	0.0296 (9)	0.0191 (7)	0.0019 (7)	0.0110 (6)	0.0039 (6)
C20	0.0325 (9)	0.0341 (10)	0.0243 (8)	0.0077 (8)	0.0195 (7)	0.0077 (7)
C21	0.0202 (7)	0.0234 (8)	0.0139 (6)	0.0012 (6)	0.0105 (5)	0.0017 (6)
C22	0.0188 (7)	0.0259 (9)	0.0162 (6)	−0.0012 (6)	0.0093 (6)	0.0004 (6)
C23	0.0247 (7)	0.0210 (8)	0.0178 (6)	−0.0019 (6)	0.0118 (6)	0.0006 (6)
C24	0.0221 (7)	0.0222 (8)	0.0156 (6)	0.0028 (6)	0.0115 (6)	0.0020 (6)
C25	0.0187 (7)	0.0239 (8)	0.0157 (6)	−0.0011 (6)	0.0080 (6)	0.0000 (6)
C26	0.0221 (7)	0.0210 (8)	0.0175 (6)	−0.0016 (6)	0.0110 (6)	−0.0006 (6)
C27	0.0216 (7)	0.0300 (10)	0.0230 (7)	0.0039 (7)	0.0095 (6)	0.0039 (7)

### Geometric parameters (Å, °)

**Table:**

S1—O3	1.4455 (13)	C11—H11A	0.9299
S1—O4	1.4602 (14)	C12—C13	1.401 (2)
S1—O2	1.4628 (12)	C12—C17	1.410 (2)
S1—C21	1.7754 (16)	C13—C14	1.382 (2)
O1—C24	1.3644 (19)	C13—H13A	0.9301
O1—C27	1.431 (2)	C14—C15	1.417 (2)
O1W—H1W	0.8450	C14—H14A	0.9297
O1W—H2W	0.8529	C15—C16	1.415 (2)
N1—C9	1.353 (2)	C16—C17	1.380 (2)
N1—C1	1.411 (2)	C16—H16A	0.9299
N1—C20	1.470 (2)	C17—H17A	0.9299
N2—C15	1.368 (2)	C18—H18A	0.9600
N2—C18	1.453 (2)	C18—H18B	0.9600
N2—C19	1.455 (2)	C18—H18C	0.9600
C1—C2	1.388 (2)	C19—H19A	0.9600
C1—C6	1.418 (2)	C19—H19B	0.9600
C2—C3	1.365 (3)	C19—H19C	0.9600
C2—H2A	0.9299	C20—H20A	0.9600
C3—C4	1.402 (3)	C20—H20B	0.9600
C3—H3A	0.9301	C20—H20C	0.9600
C4—C5	1.365 (3)	C21—C26	1.387 (2)
C4—H4A	0.9300	C21—C22	1.398 (2)
C5—C6	1.429 (2)	C22—C23	1.382 (2)
C5—H5A	0.9301	C22—H22A	0.9300
C6—C7	1.409 (2)	C23—C24	1.399 (2)
C7—C8	1.358 (2)	C23—H23A	0.9301
C7—H7A	0.9300	C24—C25	1.390 (2)
C8—C9	1.433 (2)	C25—C26	1.393 (2)
C8—H8A	0.9301	C25—H25A	0.9300
C9—C10	1.450 (2)	C26—H26A	0.9301
C10—C11	1.350 (2)	C27—H27A	0.9600
C10—H10A	0.9299	C27—H27B	0.9600
C11—C12	1.447 (2)	C27—H27C	0.9600
			
O3—S1—O4	113.13 (9)	C13—C14—H14A	119.6
O3—S1—O2	113.11 (8)	C15—C14—H14A	119.6
O4—S1—O2	112.31 (8)	N2—C15—C16	121.37 (14)
O3—S1—C21	106.25 (7)	N2—C15—C14	121.43 (14)
O4—S1—C21	105.80 (7)	C16—C15—C14	117.20 (14)
O2—S1—C21	105.44 (8)	C17—C16—C15	121.17 (14)
C24—O1—C27	118.33 (14)	C17—C16—H16A	119.4
H1W—O1W—H2W	109.3	C15—C16—H16A	119.4
C9—N1—C1	122.94 (14)	C16—C17—C12	121.62 (15)
C9—N1—C20	119.79 (14)	C16—C17—H17A	119.2
C1—N1—C20	117.24 (14)	C12—C17—H17A	119.2
C15—N2—C18	120.55 (14)	N2—C18—H18A	109.5
C15—N2—C19	120.40 (14)	N2—C18—H18B	109.5
C18—N2—C19	118.91 (14)	H18A—C18—H18B	109.5
C2—C1—N1	122.11 (15)	N2—C18—H18C	109.5
C2—C1—C6	120.29 (16)	H18A—C18—H18C	109.5
N1—C1—C6	117.60 (15)	H18B—C18—H18C	109.5
C3—C2—C1	119.21 (17)	N2—C19—H19A	109.5
C3—C2—H2A	120.4	N2—C19—H19B	109.5
C1—C2—H2A	120.4	H19A—C19—H19B	109.5
C2—C3—C4	121.91 (18)	N2—C19—H19C	109.5
C2—C3—H3A	119.0	H19A—C19—H19C	109.5
C4—C3—H3A	119.1	H19B—C19—H19C	109.5
C5—C4—C3	120.31 (18)	N1—C20—H20A	109.5
C5—C4—H4A	119.8	N1—C20—H20B	109.5
C3—C4—H4A	119.8	H20A—C20—H20B	109.5
C4—C5—C6	119.22 (16)	N1—C20—H20C	109.5
C4—C5—H5A	120.5	H20A—C20—H20C	109.5
C6—C5—H5A	120.3	H20B—C20—H20C	109.5
C7—C6—C1	119.76 (16)	C26—C21—C22	119.36 (15)
C7—C6—C5	121.21 (16)	C26—C21—S1	120.01 (13)
C1—C6—C5	119.03 (16)	C22—C21—S1	120.63 (11)
C8—C7—C6	120.31 (16)	C23—C22—C21	120.46 (14)
C8—C7—H7A	119.8	C23—C22—H22A	119.7
C6—C7—H7A	119.9	C21—C22—H22A	119.8
C7—C8—C9	121.03 (16)	C22—C23—C24	119.73 (16)
C7—C8—H8A	119.5	C22—C23—H23A	120.1
C9—C8—H8A	119.5	C24—C23—H23A	120.2
N1—C9—C8	118.18 (15)	O1—C24—C25	124.66 (14)
N1—C9—C10	120.42 (14)	O1—C24—C23	115.07 (15)
C8—C9—C10	121.40 (15)	C25—C24—C23	120.28 (15)
C11—C10—C9	123.04 (15)	C24—C25—C26	119.41 (14)
C11—C10—H10A	118.5	C24—C25—H25A	120.3
C9—C10—H10A	118.5	C26—C25—H25A	120.3
C10—C11—C12	126.36 (15)	C21—C26—C25	120.74 (16)
C10—C11—H11A	116.8	C21—C26—H26A	119.6
C12—C11—H11A	116.9	C25—C26—H26A	119.7
C13—C12—C17	117.13 (15)	O1—C27—H27A	109.5
C13—C12—C11	119.28 (14)	O1—C27—H27B	109.5
C17—C12—C11	123.58 (15)	H27A—C27—H27B	109.5
C14—C13—C12	121.99 (15)	O1—C27—H27C	109.5
C14—C13—H13A	119.0	H27A—C27—H27C	109.5
C12—C13—H13A	119.0	H27B—C27—H27C	109.5
C13—C14—C15	120.88 (15)		
			
C9—N1—C1—C2	−175.88 (16)	C12—C13—C14—C15	0.3 (2)
C20—N1—C1—C2	5.9 (2)	C18—N2—C15—C16	−179.39 (16)
C9—N1—C1—C6	4.6 (2)	C19—N2—C15—C16	5.0 (2)
C20—N1—C1—C6	−173.66 (15)	C18—N2—C15—C14	0.4 (2)
N1—C1—C2—C3	−178.39 (16)	C19—N2—C15—C14	−175.14 (15)
C6—C1—C2—C3	1.1 (3)	C13—C14—C15—N2	−179.73 (15)
C1—C2—C3—C4	0.4 (3)	C13—C14—C15—C16	0.1 (2)
C2—C3—C4—C5	−1.1 (3)	N2—C15—C16—C17	179.42 (15)
C3—C4—C5—C6	0.3 (3)	C14—C15—C16—C17	−0.4 (2)
C2—C1—C6—C7	178.90 (16)	C15—C16—C17—C12	0.4 (2)
N1—C1—C6—C7	−1.6 (2)	C13—C12—C17—C16	0.0 (2)
C2—C1—C6—C5	−1.9 (2)	C11—C12—C17—C16	179.05 (15)
N1—C1—C6—C5	177.59 (14)	O3—S1—C21—C26	−97.34 (14)
C4—C5—C6—C7	−179.62 (17)	O4—S1—C21—C26	23.18 (15)
C4—C5—C6—C1	1.2 (2)	O2—S1—C21—C26	142.35 (13)
C1—C6—C7—C8	−1.9 (2)	O3—S1—C21—C22	81.83 (14)
C5—C6—C7—C8	178.93 (16)	O4—S1—C21—C22	−157.66 (13)
C6—C7—C8—C9	2.7 (3)	O2—S1—C21—C22	−38.48 (15)
C1—N1—C9—C8	−3.9 (2)	C26—C21—C22—C23	0.8 (2)
C20—N1—C9—C8	174.28 (15)	S1—C21—C22—C23	−178.35 (12)
C1—N1—C9—C10	176.41 (14)	C21—C22—C23—C24	−0.2 (2)
C20—N1—C9—C10	−5.4 (2)	C27—O1—C24—C25	3.9 (2)
C7—C8—C9—N1	0.2 (2)	C27—O1—C24—C23	−175.95 (14)
C7—C8—C9—C10	179.86 (16)	C22—C23—C24—O1	179.57 (14)
N1—C9—C10—C11	170.96 (15)	C22—C23—C24—C25	−0.3 (2)
C8—C9—C10—C11	−8.7 (2)	O1—C24—C25—C26	−179.62 (14)
C9—C10—C11—C12	−177.83 (15)	C23—C24—C25—C26	0.2 (2)
C10—C11—C12—C13	−177.82 (16)	C22—C21—C26—C25	−0.9 (2)
C10—C11—C12—C17	3.2 (3)	S1—C21—C26—C25	178.29 (12)
C17—C12—C13—C14	−0.3 (2)	C24—C25—C26—C21	0.4 (2)
C11—C12—C13—C14	−179.40 (15)		

### Hydrogen-bond geometry (Å, °)

**Table:**

*D*—H···*A*	*D*—H	H···*A*	*D*···*A*	*D*—H···*A*
O1W—H1W···O2^i^	0.84	2.04	2.875 (3)	169
O1W—H2W···O4^ii^	0.85	2.10	2.926 (2)	161
C7—H7A···O3^iii^	0.93	2.49	3.015 (3)	116
C8—H8A···O3^iii^	0.93	2.57	3.049 (3)	113
C20—H20A···O4^iv^	0.96	2.46	3.325 (2)	151
C23—H23A···O1W^v^	0.93	2.44	3.365 (2)	176
C26—H26A···O4	0.93	2.56	2.921 (2)	104
C27—H27A···O1W^vi^	0.96	2.58	3.160 (3)	119
C27—H27A···O1^vii^	0.96	2.55	3.282 (2)	133
C16—H16A···Cg1^iv^	0.93	2.81	3.6513 (19)	151

Symmetry codes: (i) −*x*+1, −*y*, −*z*+1; (ii) *x*, −*y*+1/2, *z*−1/2; (iii) −*x*+1, *y*+1/2, −*z*+3/2; (iv) −*x*+1, −*y*+1, −*z*+1; (v) *x*, −*y*−1/2, *z*+1/2;  (vi) −*x*+2, *y*−1/2, −*z*+3/2; (vii) −*x*+2, −*y*−1, −*z*+2.

## References

[bb16] Allen, F. H. (2002). *Acta Cryst.* B**58**, 380–388. 10.1107/s0108768102003890 12037359

[bb1] Allen, F. H., Kennard, O., Watson, D. G., Brammer, L., Orpen, A. G. & Taylor, R. (1987). *J. Chem. Soc. Perkin Trans. 2*, pp. S1–S19.

[bb2] Bernstein, J., Davis, R. E., Shimoni, L. & Chang, N.-L. (1995). *Angew. Chem. Int. Ed. Engl.* **34**, 1555–1573.

[bb3] Bruker (2005). *APEX2*, *SAINT* and *SADABS* Bruker AXS Inc., Madison, Wisconsin, USA.

[bb4] Chantrapromma, S., Jindawong, B., Fun, H.-K. & Patil, P. S. (2007*a*). *Anal. Sci.* **23**, x81–x82.

[bb5] Chantrapromma, S., Jindawong, B., Fun, H.-K. & Patil, P. S. (2007*b*). *Acta Cryst.* E**63**, o2124–o2126.

[bb6] Chantrapromma, S., Jindawong, B., Fun, H.-K., Patil, P. S. & Karalai, C. (2006). *Acta Cryst.* E**62**, o1802–o1804.

[bb7] Chantrapromma, S., Jindawong, B., Fun, H.-K., Patil, P. S. & Karalai, C. (2007). *Anal. Sci.* **23**, x27–x28.

[bb8] Chia, W.-L., Chen, C.-N. & Sheu, H.-J. (1995). *Mater. Res. Bull.* **30**, 1421–1430.

[bb9] Dittrich, Ph., Bartlome, R., Montemezzani, G. & Günter, P. (2003). *Appl. Surf. Sci.* **220**, 88–95.

[bb10] Jindawong, B., Chantrapromma, S., Fun, H.-K. & Karalai, C. (2005). *Acta Cryst.* E**61**, o3237–o3239.

[bb11] Nogi, K., Anwar, U., Tsuji, K., Duan, X.-M., Okada, S., Oikawa, H., Matsuda, H. & Nakanishi, H. (2000). *Nonlinear Optics*, **24**, 35–40.

[bb12] Otero, M., Herranz, M. A., Seoane, C., Martín, N., Garín, J., Orduna, J., Alcalá, R. & Villacampa, B. (2002). *Tetrahedron*, **58**, 7463–7475.

[bb13] Sato, N., Rikukawa, M., Sanui, K. & Ogata, N. (1999). *Synth. Met.* **101**, 132–133.

[bb14] Sheldrick, G. M. (2008). *Acta Cryst.* A**64**, 112–122. 10.1107/S0108767307043930 18156677

[bb15] Spek, A. L. (2003). *J. Appl. Cryst.* **36**, 7–13.

